# Recent Foreign Investigations of Swine Diseases

**Published:** 1891-09

**Authors:** Leonard Pearson


### RECENT FOREIGN INVESTIGATIONS OF SWINE DISEASES.

By Leonard Pearson, B. S., V. M. D.

Several German bacterialogists have recently investigated the various contagious diseases of swine, and the group of bacteria to which the causes of these diseases belong.

Frosch, in an article in the *Zeitschrift für Hygiene*, Vol. ix, 1891, makes the following statements: "According to Salmon, there are two distinct contagious diseases of swine in America, which he designates as "Swine-plague" and "Hog-cholera." This conclusion has been denied by Frank Billings, who doubts the etiological value of Salmon's bacteria, and claims that a germ discovered by him is the cause of the American swine diseases; which he describes together, as one disease, under the name "Swine-plague."

In 1888 Von Esararch studied, in the Institute of Hygiene, in Berlin, the Salmon hog-cholera bacillus and found that Salmon's statements were in the main, correct. Frosch has now studied the Billing's swine-plague bacillus, cultures of which were sent to the Institute of Hygiene, by the discoverer, and finds it to be identical with Salmon's hog-cholera bacillus. The microbe described by Salmon as the cause of *his* swine-plague, is regarded by Frosch, on the other hand, as of accidental occurrence in chronic hog-cholera.

The entire article is quite long, but the author has thus concisely stated his conclusions:

1st. Salmon's hog-cholera bacillus and Billing's swine-plague bacillus are one and the same.

2d. This germ is to be regarded as the cause of the contagious disease existing among the swine of America, while the proof of the existence of a second disease, of equal distribution, is not, as yet, established.

3d. This germ is, further, identical with the swine-pest bacillus of Selander's (Denmark), but differs from the bacillus of the German swine-plague from that of Wildseuche (a contagious disease of deer, wild hogs, and cattle, which occurs in South Germany), chicken-cholera, rabbit septicæmia, and the ferret plague.

4th. Of those last mentioned, the bacterian of the ferret plague is the only one which possesses peculiarities sufficiently pronounced to enable its recognition from others. (*Berl. Thierarz. Wochs., No.* 27, 1891.)

Dr. Georg Caneva (Centralblatt für Bacteriologie und Parasitenkunde, Vol. ix, No. 17), studied the group of bacteria which have been classed together by Hueppe, as those causing hemorrhagic septicæmia. These are: hog-cholera (Salmon), swine-plague (Billings), swine-pest (Selander), "American cattle disease" (Billings), Buffalo pest Marseille's swine-pest (Jobert, Rietsch), and the ferret plague (Eberth). All of these bacteria agree in the following points: they liquify gelatine; do not form end spores; the poles may be stained with an aqueous solution of methyline blue but not by Graue's method; and there is a resemblance in form. Notwithstanding their apparent close relationship, the author found differences which enabled him to divide this group into three subdivisions.

The first includes the bacteria of Wildseuche, septicæmia of rabbits, swine-plague (of Germany) and Buffalo pest. These bacteria are characterized by lack of mobility, by the fact that they will not grow on potatoes, their growth on gelatine and on agar-agar is slow and sparse, they grow scantily on milk and kill rabbits in 1-3 days after the inoculation. The autopsy shows them to be present in the blood and distributed through the tissues.

The second division includes swine-plague (Billings), Marseilles swine pest (Epidemie des Porcs de Marseille), the "new American cattle disease" (Billings), and the ferret plague. These bacteria are distinguished by active mobility; they grow rapidly on potato and form a thick layer, but differ among themselves in the color of the film. They grow rapidly on gelatine and agar-agar and, on the latter, with the development of gas. Cultures in milk, at the incubator temperature (37°C.), develop an acid which coagulates the milk, but does not cause the solution of its suspended particles. White rats and mice are not susceptible to these bacteria and, upon being inoculated, only show a slight local reaction at the point of operation. The bacteria may be found in the blood and in the form of small emboli, in the capillaries, but never in the tissues themselves.

The third division consists of only the hog-cholera (Salmon), and swine pest (Selander) bacilli. These are not exactly alike, but show so many points of similarity that it is difficult to separate them. Both are actively mobile; they grow rapidly and profusely on gelatine. They also grow on agar-agar but without the formation of gas. They differ in their growth on potatoes, in that the swine-pest cultures resemble those of the typhus bacillus, while the hog-cholera bacilli forms a thick, whitish layer. Hog-cholera bacilli kills rabbits in from 4-8 days and cause a reaction at the point of inoculation. Subcutaneous injections of the swine-pest bacilli, on the other hand, do not kill rabbits but do white mice, in from 6-8 days.

Caneva's deductions from his experiments are: that Salmon's hog-cholera and Billing's swine-plague are two distinct diseases; that Billing's swine-plague and the Marseilles swine pest are, probably, identical; that, although Selander's (Denmark) swine pest and hog-cholera are closely related they are distinct diseases and, finally, that none of the above diseases coincide with the German swine-plague of Löffler and Schütz's. (*Zeitsch. für Vet. Kund.*, No. 4. Vol. iii, 1891.)

So far as the diseases of our swine are concerned it will be noticed that the two investigators, above quoted, do not agree. It is, however, highly probable that they were not working with the same sort of material.

The following extract, from an article by Dr. E. Bunzl-Fedem (*Archiv für Hygiene*, Vol. xii, Heft 2), throws some light on this point. This long article gives the results of the author's studies of the members of the septicæmia-haemorrhagica group of bacilli, but since a review of it as a whole would occupy too much space we shall only mention the portion that deals with the diseases of swine. Cultures of the swine disease bacilli were made in sterilized milk, of neutral reaction, to which a small quantity of a litmus solution had been added, to show fermentation at its beginning, and on potato. Bacilli of swine-plague (Billings), and hog-cholera (Salmon), both grew in the sterilized milk, turning the litmus faintly red, in the first two days, but intensively blue in from eight to ten days. After seven months the milk had so evaporated that it formed a gelatine-like mass, of alkaline reaction. Both of these bacilli formed brown films on potatos. The German and Marseilles swine pests, on the contrary, developed an acid in the milk cultures.

From these and other experiments the deductions drawn are:

1st. That the swine-plague germ first cultivated by Billings does not agree with the description of that micro-organism.

2d. That Salmon's hog-cholera bacillus and the the last swine-plague bacillus cultivated by Billings are, biologically, identical.

Detailed experiments lead him to divide the contagious swine-diseases of the world into three classes, as follows: 1st. German swine pest and wildseuche; 2d. Swine-plague, hog-cholera and Danish swine pest; 3d. Marseilles swine pest.

These diseases differ in their specific causes; which, in spite of their strong similarity, may be differentiated biologically in their symptoms and in their geographical origins.

Bunzl-Fedem believes that the future will develop methods for diagnosing the doubtful cases of swine diseases by means of the cultures and biological peculiarities of their bacteria. The gelatine cultures enables one to distinguish all three of the above diseases from erysipelas (*Rothlauf, rouget*) or anthrax. Milk cultures distinguish the American swine disease from the Marseilles and German, in that the first develops an alkali and the last two an acid. A microscopic examination suffices to separate the last two forms, for the bacilli of the French pest are actively mobile, while those of the German have no motion. (*Berl. Thierârz. Wochs.*, *No. 29, 1891.*

From these, and other articles, it is quite clear that the most prevalent opinion in Germany, at the present time, is that there is but one swine pest in America; that is, that hog-cholera and swine-plague are the same.

Variations from this view probably rest upon experiments with those cultures of Billing's swine-plague bacillus first sent to Europe, by this investigator; which did not, Bunzl-Fedem says, agree with their description and were not the same as those sent at a later date.

